# Improvement of Fatigue and Body Composition in Women with Long COVID After Non-Aerobic Therapeutic Exercise Program

**DOI:** 10.3390/jpm15060217

**Published:** 2025-05-26

**Authors:** María Miana, Ricardo Moreta-Fuentes, Carmen Jiménez-Antona, César Moreta-Fuentes, Sofía Laguarta-Val

**Affiliations:** 1 Faculty of Nursing and Physiotherapy Salus Infirmorum, Pontifical University of Salamanca, 28015 Madrid, Spain; mmianaor@upsa.es; 2 Fisyos Center, Método Moreta, 28027 Madrid, Spain; metodomoreta@gmail.com; 3 Department of Physical Therapy, Occupational Therapy, Rehabilitation and Physical Medicine, Faculty of Health Sciences, Universidad Rey Juan Carlos (URJC), 28922 Madrid, Spain; sofia.laguarta@urjc.es; 4 FREMAP Care Center, 28946 Madrid, Spain; mofuce@gmail.com

**Keywords:** body fat distribution, exercise therapy, fatigue, post-acute COVID-19 syndrome

## Abstract

**Background/Objective**: Fatigue is one of the most recurrent and most disabling symptoms of long COVID (LC) and is associated with a worse quality of life. Reducing body fat in these patients could be important to mitigate fatigue and post-exertional worsening. Aerobic exercise may not be indicated in LC patients who have orthostatic tachycardia and post-exertional worsening. The aim of this study was to evaluate the effects of a personalized supine therapeutic motor control exercise program on fatigue and fat tissue in women with LC. **Methods**: A single-arm exploratory case study, with a pre–post format, was conducted on 17 women with LC to test the effects of a plank-based strengthening exercise program on fatigue, which was evaluated by the Modified Fatigue Impact Scale and fat tissue assessed by bioimpedance. The twelve-week program included two weekly sessions. The exercise program was personalized, considering the symptoms and characteristics of the patients. **Results**: Participants with overweight or obesity (n = 12) comprised 70% of the entire sample. After completing the exercise program this value decreased by 5.9 percentage points. Significant differences were found in the total [(MD  = −1.72, 95% CI −2.57 to −0.86), r = 0.73], trunk, upper and inner limbs body fat percentages (*p* < 0.05). The overall fatigue decreased at 12 weeks [(MD  =  −14.00, 95% CI −21.69 to −6.31), r = 0.69] as well as the physical and psychosocial fatigue sub-scale (*p*  <  0.001); no differences were observed in the cognitive sub-scale. **Conclusions**: The plank-based personalized strengthening exercise program showed rapid improvements in fatigue and fat percentages. It could be an effective strategy to achieve improvements for LC patients.

## 1. Introduction

Long COVID (LC) can involve multiple organs and can affect many systems, including, but not limited to, the respiratory, cardiovascular, neurological, gastrointestinal, and musculoskeletal systems. The symptoms of LC include fatigue, dyspnea, cardiac abnormalities, cognitive impairment, sleep disturbances, symptoms of post-traumatic stress disorder, muscle pain, concentration problems, and headaches [1]

Fatigue is one of the most recurrent and most disabling symptoms of LC and is usually persistent [2] and associated with a worse quality of life [3]. Symptoms may arise after the recovery from an acute episode of COVID-19, persist from the onset of the disease, or vary over time, fluctuating or reappearing [4]. A systematic review evaluating LC fatigue reported a prevalence rate of 34.9% of recovered cases the first six months after the infection [5]. Post-COVID-19 fatigue in previously hospitalized survivors tends to have a slow recovery that could last several years after the infection [6]. LC fatigue is thought to have a multifactorial etiology, including, among others, a persistent immune response, chronic inflammation, impacts on the central nervous system, or mitochondrial dysfunction [7]. Fatigue has been associated with different potential risk factors like an advanced aged and female sex, the severity of the disease, the duration of the acute phase or of the recovery, pre-existing autoimmune diseases, depression, or comorbidities [8,9]. Post-COVID-19 syndrome patients presented functional connectivity changes, characterized by hypoconnectivity between left and right parahippocampal areas and between bilateral orbitofrontal and cerebellar areas compared to controls. These alterations were accompanied by a reduced gray matter volume in cortical, limbic, and cerebellar areas and alterations in the white matter axial and mean diffusivity. Gray matter volume loss showed significant associations with cognitive dysfunction. The patients with LC showed persistent structural and functional brain abnormalities 11 months after the acute infection [10].

It is known that exercise could improve white matter integrity, enhance neuroplasticity, and alter cortical excitability, which contributes to the improved connectivity between brain hemispheres observed with regular exercise [11,12,13,14,15].

Several studies highlight the complexity of exercise intolerance in patients with LC. Among individuals with symptoms consistent with LC deconditioning—as well as cardiac and ventilatory limitations, such as reduced stroke volume augmentation, chronotropic incompetence, dysfunctional breathing, hyperventilation, or ventilatory inefficiency—an impaired pEO2 and a decrease in peak VO2 have been observed and may contribute to exertional intolerance [16,17,18].

The most commonly reported symptoms of LC also include a limited exercise tolerance [19] and post-exertional malaise [20], which is characterized by a worsening of fatigue- and pain-related symptoms after acute mental or physical exercise. This phenomenon may be driven by a peripheral impairment in the skeletal muscle metabolism, including a reduced oxidative phosphorylation capacity [21]. The complex interaction between physical activity and LC, the contradictory recommendations of healthcare professionals [22], or the insufficient guidance on how to resume physical activity [23] have led individuals with LC to engage in physical activity at an unsuitable intensity, worsening their symptoms. Currently, and considering the presence of orthostatic tachycardia in these individuals, the American Society of Cardiology recommend decubitus exercise as a return-to-training recommendation for athletes who have suffered from COVID-19 infections prior to their progression to standing training [24].

Fatigue has been associated with different potential risk factors, like an advanced aged and female sex, the severity of the disease, the duration of the acute phase or of the recovery, pre-existing autoimmune diseases, depression, or comorbidities [8,9]. Patients under 60 years old with a visceral adiposity or high intramuscular fat deposition had higher risks for critical illness. Yang et al. studied 45 critically ill patients out of 143 who were hospitalized [25]. Those who had a visceral adiposity or high intramuscular fat (IMF) deposition increased the risk of mechanical ventilation. And those with greater IMF had a higher risk of death.

The fat distribution and the differences in body compositions led to different COVID-19 outcomes [26]. The adipose tissue distribution has been considered as an important factor in the body’s response to infections [27].

Abdominal obesity alters pulmonary functions by diminishing the physical activity capacity and increasing the airway resistance, resulting in the increased work of breathing [28]. Lung activity is dependent on the expansion of the chest cavity and intra-abdominal cavity. The respiratory capacity of the lungs could be restricted by a mechanical pressure generated by an accumulation of adipose tissue around the chest and in the peritoneal cavity [29]. Visceral fat depots were proven to increase the risk for severe COVID-19, being in part responsible for restricting diaphragm movements and limiting lung activity and function [30].

We hypothesized that a personalized plank-based therapeutic exercise program, supervised by physiotherapists, with a total of 24 sessions, twice a week, for three months could reduce fat tissue and could reduce the self-perceived fatigue in a group of women affected by LC.

## 2. Methods

### 2.1. Design

A single-arm non-blinded exploratory case study, with a pre–post format was carried out with women with LC to test the effects of a plank-based strengthening exercise program on fat tissue and fatigue. This study has been reviewed and approved by Hospital Foundation Alcorcon’s ethics committee under the number 21/173. No substantial changes in methods were made after the start of the trial.

### 2.2. Participants

Recruitment took place at the clinic of the Faculty of Health Sciences of the Rey Juan Carlos University. Patients from the LC association of Madrid (AMACOP) applied to an email address created for this purpose, managed by the principal investigator of the project, a full professor at the Universidad Rey Juan Carlos. Through this email address, patients resulting from non-probability consecutive sampling received further information and documentation about the trial and were summoned by telephone to the office of the Rehabilitation Doctor (doctor at the Ramón y Cajal public hospital and part-time professor at the Rey Juan Carlos University) who carried out the screening.

Participants were chosen by consecutive non-probability sampling from Madrid LC association (AMACOP). All subjects met the following inclusion criteria: (1) LC symptoms for more than a year; (2) age over 18 years; and (3) complete vaccination schedule for COVID-19 according to the criteria of the Spanish Ministry of Health. Participants were excluded if they had any of the following conditions: severe heart disease, abdominal hernia, pregnancy, history of musculoskeletal injuries or surgery in the last year, and no coexistence of neuromuscular pathology. Screening started in January 2022, and the last patient exit occurred in May 2022. Written informed consent was obtained before all procedures, carried out in accordance with the principles of the Declaration of Helsinki and the Biomedical Research Act 14/2007.

### 2.3. Procedure

The participants were evaluated at the Faculty of Health Sciences of the Rey Juan Carlos University, Spain. The bioimpedance was registered before and after the proposed intervention.

Before the bioimpedance analysis, physical characteristics and other data were taken, including sex, age, weight, height, time with symptoms, hospitalization, re-infections, and other pathologies. Body mass index (BMI) was computed as weight (kg) divided by height squared (m^2^), and the rationale for classification of BMI categories was the criteria recommended by the OMS [31,32]. The Modified Fatigue Impact Scale (MFIS) was used to evaluate the participants’ fatigue before and after the described intervention, which is a widely used scale to assess fatigue in LC, being a shorter version of the FIM, consisting of 21 items instead of 40 [33]. It assesses perceived fatigue in the last 4 weeks. Physical sub-scale can range from 0 to 36, cognitive sub-scale can range from 0 to 40, psychosocial sub-scale can range from 0 to 8, and overall fatigue can range from 0 to 84. Most studies use a score of 38 as the cut-off point [34].

Segmental body composition was assessed by bioimpedance analysis (BIA) (Tanita BC-545N). In order to ensure reliability of BIA measurements, patients were recommended not to practice vigorous exercise 24 h before the measurement and to urinate.

### 2.4. Intervention

The participants performed a therapeutic exercise program based on correct body alignment and normal biomechanics, avoiding compensatory movements (MORETA program). All participants maintained their usual dietary habits throughout the intervention period.

The program consisted of 2 60 min sessions per week on alternate days, with a total of 24 sessions.

**Figure 1:**
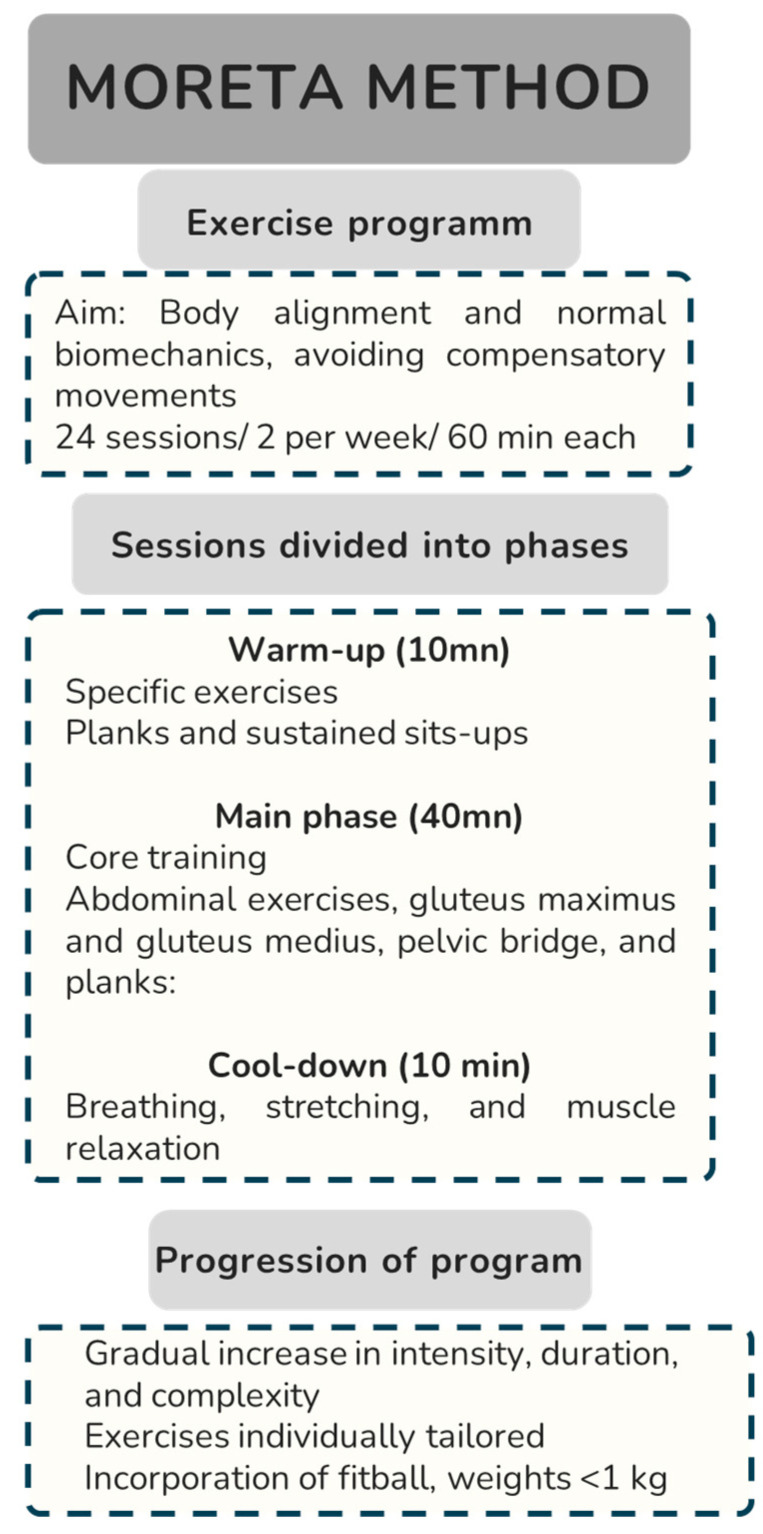
The flowchart of the Moreta Method.

Participants undertook a trunk stabilization program based on plank exercises. During the trunk musculature stabilization program, muscles and neuromuscular control are trained [35] according to the Moreta methodology (Figure 1): “The maintenance of maximum contraction, maintained for as long as possible, of the abdominals and stabilizing muscles, during the performance of all the exercises of the session, reprograms our nervous system, to increase muscle tone (Sustained and involuntary contraction, controlled by involuntary CNS centers (cerebellum, pons, etc.) This increase in tone allows for greater muscle performance due to the increase in the recruitment of motor units in each session and, in addition, trains the individual so that, involuntarily, in their daily activities, they activate that stabilizing musculature”.

These exercises can increase power, strength, balance, and proprioception [2].

Participants were separated into two subgroups for better teaching of the exercises. Each group consisted of up to 8–9 patients, supervised by 2 physiotherapists during all sessions.

The control of possible trade-offs was fully monitored. Accommodations were made for patients who needed them, for example, one who had knee arthrodesis. If the goal of the exercise was, for example, to hold a plank for one minute, the patient who could not complete it all the time could adapt it by resting their knees on the floor, or it was not necessary to complete the entire time of the exercise during the first sessions.

Each session included 10 min of warm-up (3 min of specific exercise, sitting on the wall, 1 min of anterior plank on elbows and feet, 50 sit-ups, and 1 min of sustained sit-ups), 40 min of trunk musculature training, and 10 min of cool-down (breathing, stretching, and muscle relaxation). All sessions included abdominal exercises, gluteus maximus and gluteus medius, pelvic bridge (according to the Moreta Method) and planks: anterior plank on elbows or on hands with arms straight and side plank.

The participants progressed as follows: in the first sessions they performed 150 to 200 sit-ups and at the end of the study they achieved between 300 and 400 repetitions distributed throughout the class.

We incorporated more complex exercises each week, which included leg or arm exercises combined with abdominal exercise, with the aim of increasing tone and proprioception and learning the new sequence of contractions involved in stabilizing the trunk.

In the pelvic bridge, maintenance went from 5 min to 9 min, while alternating exercises with sets that evolved from 10 to 25 repetitions. In gluteus medius exercises they spent 2 min to 4 min of maintenance and while maintaining proper posture, they were able to increase the repetitions from 20 to 60 repetitions on each side. During plank exercises, they held the position for 15 to 30 s up to 60 s.

Little by little, more complex exercises were incorporated with weights that never exceeded 1 kg and with a fit ball to increase the complexity of the exercise, thus improving the tonal component, proprioception, and the involuntary maintenance of the muscles involved in the stabilization of the trunk.

Also, depending on the objective of the class, in addition to the toning of the trunk musculature and gluteus, they alternated with classes more aimed at hips and legs, where they went from 10 initial repetitions of quadriceps splits to 50 and others aimed at shoulder girdle, where they managed to evolve from 10 plank push-ups on knees and hands to 50 repetitions on feet and hands, being able to do so while maintaining all the body parameters requested for its correct execution (In these planks and push-ups, the correct contraction of the serratus muscle and latissimus dorsi is essential to prevent movement or detach the scapula from the trunk).

In terms of intensity, a somewhat hard level of intensity was maintained [2] according to the Perceived Exertion Rating Category Scale [36].

Given the heterogeneous characteristics of LC, the exercise program was completely personalized, considering the symptoms and characteristics of the patients at the beginning of the trial.

### 2.5. Statistical Analysis

Analyses were performed using IBM SPSS version 20.0 software (IBM Corp, Armonk, NY, USA). Data were presented as mean, standard deviation, number, and percentage. Shapiro–Wilk test was used to determine whether continuous variables were normally distributed. Continuous variables were compared between groups using *t* test for paired samples. Relationships between two quantitative variables were examined using Pearson correlation analysis if normally distributed. *p* values < 0.05 were considered statistically significant.

## 3. Results

A total of 17 women with LC symptoms completed the study, and no attrition was registered during the follow-up period. The mean age was 45 (SD 7.3) years. Participants’ BMI categories at the beginning and after 12 weeks of training are presented in Table 1.

Before the program, 47% of the participants were in the overweight/pre-obesity category, and 23.5% were in class I obesity. These baseline data reveal that a significant majority of the sample population are not at their appropriate weight. After the intervention, while the percentages in the underweight and class I obesity categories decreased, the percentage of participants in the normal BMI category increased from 17.6% to 35.3%, suggesting a positive impact of the intervention, with more individuals moving towards a healthier weight range.

### 3.1. Segmental Body Composition

Significant differences were found between the total body fat (−1.72%), trunk (−2.09%), and upper and inner limb body fat before and after the intervention (*p* < 0.05) (Table 2).

The high r-values suggest that the exercise program had a significant impact on reducing the total body fat (r = 0.73), as well as in all the evaluated body segments. This indicates the program’s ability to induce consistent and significant changes in body compositions.

No differences were observed between the segmental and the total muscle mass and visceral fat level.

### 3.2. Fatigue

Participants’ fatigue before and after the described intervention are presented in Table 3.

All participants exhibited clinically significant levels of fatigue at the beginning of the study, as determined by a score greater than 38 on the MFIS. General fatigue decreased significantly after the exercise program (−14.00), with particularly notable improvements in the physical (−8.65) and psychosocial (−1.70) sub-scales. No significant changes were observed in the cognitive sub-scale.

**Figure 2:**
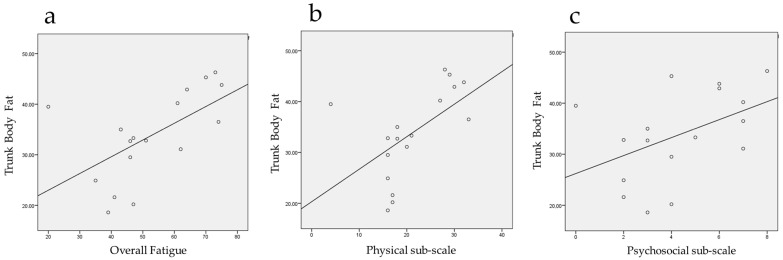
Linear correlations between trunk body fat and fatigue after training period: (**a**) overall fatigue; (**b**) physical sub-scale; and (**c**) psychosocial sub-scale.

There was a positive linear correlation between trunk body fat and overall fatigue (r = 0.591; n = 17; *p* = 0.012) and its physical (r = 0.559; n = 17; *p* = 0.020) and psychosocial (r = 0.484; n = 17; *p* = 0.049) sub-scales after the training period. These findings indicate that as participants experienced a decrease in trunk fat, their levels of fatigue also diminished (Figure 2).

## 4. Discussion

In this study, the results obtained show that 12-week plank-based strengthening exercise significantly improved fatigue, according to the MFIS, in women with LC. Moreover, the bioimpedance assessment showed that patients have also improved their proportion of total fat tissue as well as the fat tissue in their different locations—right and left upper limb, right and left inner limb, and trunk.

Fatigue is presented as the most prevalent symptom in patients with LC and is reported by 36.9–44% of patients.

Tabacof et al. [37] demonstrated that in patients with long COVID, the most common persistent symptom reported was fatigue (82%). Their results are consistent with the present results because they obtained increased levels of fatigue (Fatigue Severity Scale). The most common trigger of symptom exacerbation Tabacof established was physical exertion (86%). However, in our study we reported, not a worsening of symptoms but, an improvement of symptoms throughout the start of the trail in June 2022, subsequently after the end of the trial, and until today.

Twomey et al. [38] conducted a study involving 213 patients with LC; 71.4% of whom were experiencing chronic fatigue. The exacerbation of post-exertional symptoms affected most of the participants. No worsening of symptoms was reported in our trial.

The results are consistent with another study in which non-invasive brain stimulation paired with a rehabilitation program was effective in reducing fatigue in people with LC. The fatigue was also assessed by the MFIS [39].

The physiological mechanisms underlying the fatigue of LC patients are currently unknown, although evidence is emerging on a possible link with mental fog, a symptom also present in a large number of LC patients.

In the year in which this study was conducted, a systematic review and meta-analysis of 81 studies established that a third of patients experienced persistent fatigue and over a fifth of patients exhibited cognitive impairment 12 or more weeks following a COVID-19 diagnosis [40].

In the same year, brain changes were demonstrated in 785 participants who were imaged twice using magnetic resonance imaging. This trial obtained a greater reduction in the gray matter thickness and tissue contrast in the orbitofrontal cortex and parahippocampal gyrus; greater changes in markers of tissue damage in regions that are functionally connected to the primary olfactory cortex; and a greater reduction in the global brain size in the SARS-CoV-2 cases, relative to the control group [41].

Only a year later, Ortelli et al. [42] showed objective reductions in cognitive performance and higher fatigability as compared to the control group. These patients, at the same time, showed a reduction in delta source power. That reduction was found bilaterally in the frontal–parietal lobe network and in the left temporal lobe; these are brain regions involved in the central executive network, default mode network, salience network, and sensorimotor network (SMN).

Some authors have linked the possible dysfunction of the motor cortex to the fatigue experienced by patients with LC.

A total of 67 post-COVID-19 patients were evaluated utilizing the transcranial magnetic stimulation of the primary motor cortex (M1) and analyzed the resting motor threshold, motor-evoked potential amplitude, cortical silent period duration, short-interval intracortical inhibition, intracortical facilitation, long-interval intracortical inhibition, and short-latency afferent inhibition. Patients with fatigue and cognitive difficulties following mild COVID-19 presented an altered excitability and neurotransmission within the M1 and deficits in executive functions and attention. [43].

Casula et al [44]. used transcranial magnetic stimulation combined with EEG (TMS-EEG) to explore the neural oscillatory activity of the left primary motor area (l-M1) and supplementary motor area (SMA) in a group of sixteen post-COVID patients complaining of lingering fatigue as compared to a sample of age-matched healthy controls. Perceived fatigue was assessed with the Fatigue Severity Scale (FSS) and the Fatigue Rating Scale (FRS). Post-COVID patients showed a remarkable reduction in beta frequency in both areas. They demonstrated that post-COVID-19 fatigue is associated with a reduction in the TMS-evoked beta oscillatory activity in the SMA.

We hypothesize that the combination of anti-inflammatory effects, increased neurotrophic support, enhanced neuroplasticity, and improved physical health through exercise can contribute to the reduction in TMS-evoked beta oscillatory activity in the SMA and the improved connectivity of the motor cortex in LC patients.

Firstly, exercise is known to exert positive effects on brain health by modulating inflammatory markers, neurotransmitters, and neurotrophic factors, which are pathways disrupted by COVID-19 infections [45]. This modulation can help restore normal brain function and reduce neuropsychological symptoms associated with LC. Secondly, aerobic exercise has been shown to increase brain-derived neurotrophic factor (BDNF) levels, which supports neuroplasticity and synaptic efficacy [46]. This increase in BDNF can enhance the brain’s ability to adapt and reorganize, potentially improving the connectivity and function in the motor cortex. Thirdly, exercise can reduce GABAergic inhibition and increase corticospinal excitability, as demonstrated by studies using TMS [47]. This reduction in inhibition and increase in excitability can facilitate better motor control and connectivity, which may be particularly beneficial for patients experiencing motor deficits due to LC. Lastly, exercise has been shown to improve the overall cardiorespiratory and muscular function, which can indirectly support better brain function by enhancing overall physical health and reducing fatigue [48].

In relation to fatigue, it was observed that after the 2-month program, the patient sample improved significantly compared to the baseline. Persistent fatigue, 16–20 weeks after the symptom onset, is a symptom present in 13–33% of COVID-19 infected persons [49]. The impact of fatigue symptoms on functionality in people with LC has been evaluated, showing that 15–40% of patients are unable to return to work 2–4 months after the onset of symptoms [38]. Given the importance and prevalence of fatigue among people diagnosed with LC, it is relevant to highlight the importance of establishing exercise programs that allow people with LC to reduce their fatigue levels (assessed in this study with the MFIS) and to produce an indirect improvement in self-perceived functionality.

The patients included improved their overall MFIS score, physical sub-scale score, and psychosocial sub-scale score but not their cognitive sub-scale score, which may indicate that people with LC need options in addition to exercise programs to reduce physical fatigue levels.

Di Profio et al. [50] assessed body compositions in 40 children affected by Multisystem Inflammatory Syndrome. The fat mass index was significantly higher 6 months after an acute event. Thus, limiting physical activity and having a sedentary lifestyle may lead to an accumulation of adipose tissue even in healthy children who experienced Multisystem Inflammatory Syndrome and long hospitalizations. Our trial obtained a total adherence to the program to avoid an accumulation of adipose tissue because of a sedentary lifestyle.

These results are consistent with a trial with women with obesity which studied and proved the effect of a therapeutic gymnastics program on the body weight composition of women with obesity, which indicated a reduction in body fat [51].

Reducing body fat in patients with LC is important to mitigate fatigue and post-exertional worsening due to several interconnected physiological mechanisms.

Firstly, LC is associated with mitochondrial dysfunction, which impairs the fat oxidation capacity during exercise, leading to increased fatigue and reduced physical performance [52]. Excess body fat can exacerbate this metabolic disturbance by further impairing mitochondrial function and increasing systemic inflammation, which are already prevalent in LC patients [21,53].

Secondly, greater body fat is linked to a reduced muscle strength and mass, which are critical for maintaining physical function and reducing fatigue. Patients with LC often exhibit reduced muscle strength, which is partly mediated by the lower appendicular lean mass index (ALMI) [53]. Reducing body fat can help improve muscle mass and strength, thereby enhancing the overall physical capacity and reducing fatigue.

Additionally, excess body fat contributes to systemic inflammation and oxidative stress, which are significant factors in the pathophysiology of long LC. These factors can worsen fatigue and post-exertional malaise by promoting a hypometabolic state and impairing energy production [53,54].

Therefore, reducing body fat through appropriate interventions such as exercise can help improve mitochondrial function, reduce systemic inflammation, enhance muscle strength, and ultimately alleviate fatigue and post-exertional worsening in patients with LC.

Aerobic exercise may not be indicated in patients with LC cardiovascular phenotypes who have orthostatic tachycardia and post-exertional worsening due to several reasons:

1. Orthostatic Tachycardia: Patients with postural orthostatic tachycardia syndrome (POTS) experience significant increases in their heart rate upon standing, which can be exacerbated by upright aerobic exercise. The American College of Cardiology (ACC) notes that these patients often cannot tolerate upright exercise, such as power walking or jogging, as it can worsen symptoms like fatigue and post-exertional malaise [24].
2. Post-Exertional Malaise: Post-exertional malaise (PEM) is a hallmark of LC, characterized by a significant worsening of symptoms following physical exertion. This can include increased fatigue, muscle pain, and cognitive difficulties, which can persist for days or even weeks after the activity. The National Institute for Health and Care Excellence (NICE) guidelines caution against graded exercise therapy in patients with LC, particularly those with myalgic encephalomyelitis/chronic fatigue syndrome (ME/CFS) [55].
3. Exercise Intolerance: Studies have shown that patients with LC often have a reduced exercise capacity and chronotropic incompetence, which can lead to exercise intolerance. This is associated with a lower peak oxygen consumption and muscle strength, making it difficult for these patients to engage in and benefit from traditional aerobic exercise [56]. Given these factors, it is recommended that exercise regimens for these patients be tailored to their specific needs, starting with recumbent or semi-recumbent exercises (e.g., rowing, swimming, or cycling) and gradually progressing as tolerated. This approach helps to avoid exacerbating symptoms while still promoting physical activity [24].

Our proposed program focused on fat reduction with no worsening of post-exertional symptoms. This is consistent with expert recommendations and previous clinical trials [9,57,58].

More than one in three previously healthy college athletes recovering from COVID-19 infections showed imaging features of a resolving pericardial inflammation [59].

LC is characterized by profound fatigue, impaired functional capacity with post-exertional malaise, orthostatic intolerance, and tachycardia. At least 25–30% of individuals impacted by SARS-CoV-2 will go on to experience LC syndrome, underscoring the detrimental impact this condition has on society.

Endurance interventions are recommended to improve fatigue in LC, which is also based on diseases that also produce cardiac deconditioning. Many of the exercises suggested by successful trials are decubitus supine exercises [9,60].

Edward et al. [61] suggest exercise prescriptions tailored to the LC patient based on the pathophysiology underlying this syndrome, as well as the previously demonstrated benefits of exercise training in other similar populations whose clinical manifestations result from cardiac deconditioning. Their systematic review achieved improvements in the VO2 peak and fatigue post endurance training programs with LC patients [60,61].

The Moreta program was chosen because it was designed by clinical physiotherapists with experience in the management of complex patients and because it met the requirements of resistance training and decubitus positions (with 95% of the program performed in the supine position) recommended by the available evidence for this type of patient.

A personalized supine strengthening and motor control exercise program, like the Moreta program, could improve the fatigue, fat tissue reduction, and post-exertional malaise (PEM) in LC patients through several mechanisms supported by the current evidence.

Firstly, individualized exercise programs tailored to the patient’s symptoms and fatigue levels have been shown to significantly reduce fatigue and improve the physical performance capacity in LC patients. A randomized controlled trial demonstrated that a symptom-titrated exercise program led to significant improvements in fatigue severity and the health-related quality of life [62]. This approach ensures that the exercise intensity and duration are adjusted based on the patient’s daily fatigue levels, making it a safe and effective strategy for managing fatigue in LC.

Secondly, exercise programs that include both aerobic and resistance training have been associated with reductions in fat tissue. A multidisciplinary rehabilitation program, which included physical training, showed a significant decrease in abdominal fatty tissue among LC patients [63].

Lastly, addressing post-exertional malaise is crucial in LC management. The American College of Cardiology recommends recumbent or semi-recumbent exercises for patients with exercise intolerance and PEM, as these exercises are less likely to exacerbate symptoms compared to upright exercises [9]. This aligns with the concept of supine strengthening exercises, which can help improve physical capacity without triggering PEM.

In summary, a personalized supine strengthening and motor control exercise program can effectively improve fatigue, reduce fat tissue, and manage PEM in LC patients by tailoring the exercise regimen to individual tolerance levels and focusing on safe exercise modalities.

This trial achieved reduced fat tissue like Ohkawara’s et al.’s [64] study, which evaluated one aerobic exercise program in subjects with obesity without metabolic-related disorders. However, our study obtained these results without aerobic exercise. Therefore, we consider it to be a useful tool for people suffering from LC, because 86% of them experience a worsening of their symptoms after physical exertion.

According to Lakhdar et al.’s [65] results, aerobic exercise for 6 months was found to have no effects on the circulating and abdominal adipose tissue in women with obesity. But this trial obtained the result of decreasing the trunk fat in only 3 months, with a special significance for women with obesity.

Exercise alone, without diet treatments, had a minimal impact on measured outcomes in a trial on women with obesity, which is not consistent with our findings. But exercise participation resulted in significant improvements in quality of life and body image in their trial.

With muscular fatigue and chronic fatigue, the muscle feels floppy and the force generated by muscles is always low, causing the patient to feel frail constantly. The leading cause underpinning the development of chronic fatigue is related to coronavirus disease 2019 (COVID-19) [66]. Our patients improved their frail feelings.

The severity of fatigue is influenced by a worsening quality of life, heightened anxiety levels, and decreased peripheral muscle strength. Additionally, a worse quality of life was associated with a higher sensation of dyspnea, lower muscle strength, and reduced physical capacity [67]. Our patients did not decrease their physical and psychosocial sub-scales according to the MFIS.

Cavigli et al. conducted a very useful review for exercise prescriptions after SARS-CoV-2 infections [68]. Although most of the papers they reviewed focus on hospitalized patients, our sample was chosen without hospitalization so that it could not be influenced by possible post-ICU syndromes or the sedentary lifestyle that hospitalization entails, together with what was already entailed by the confinement during the pandemic period, because our patients were first infected in the first wave of the pandemic. The difference between LC and post-COVID-19 patients is that the former suffered the same symptoms from the acute onset, which did not resolve. The latter suffered sequelae after the period of infection [69].

This review also includes recommendations for the prescription of exercise in athletes during their return to activity, which we consider very useful.

The work of Cavigli et al. [68] is very complete and presents recommendations consistent with our intervention in the following aspects:

The exercise prescription is based on the so-called ‘FITT’ model (frequency, intensity, time, and type). In terms of frequency, two times a week and in the supine or semi-recumbent position is suggested. Intensity: moderate intensity is recommended for this type of patient and the Perceived Exertion Rating Category Scale was used, as recommended by this review. Time: our session duration (60 min) and total intervention time (12 weeks) were also consistent with the recommendations of this review. The type of exercises included were resistance training, strengthening, respiratory, flexibility, and balance exercises. We also worked on the importance of diaphragmatic breathing during the performance of the exercises. This is consistent with the proposal of these authors.

We are also in line with the recommendations of the authors on the following points:

- In a safe and effective rehabilitation program, physical activity should be prescribed with a tailored approach and personalized based on individual characteristics.
- The patient should be evaluated in his/her complexity to adapt an exercise program appropriate to their characteristics, health problems, symptoms, and consequences of COVID-19.
- A multidisciplinary team should establish a general treatment plan adapted for each patient according to the clinical presentation [68].

Some factors could compromise the external validity of this study: some groups are underrepresented and there are a lot of independent variables associated with the LC condition that could influence the obtained results, making the research irreplicable in other situations.

This trial has some limitations such as the absence of a control group or the lack of a comparison with a second intervention of a different physical exercise.

Future lines of research should be considered in order to determine the optimal sample size and assure an adequate power to detect statistical significance compared to a control group. Likewise, is important to explore similar effects in male sample with LC.

## 5. Conclusions

A plank-based strengthening exercise program showed rapid improvements in fatigue and fat percentages. It could be an effective strategy to improve the state of LC patients.

## Figures and Tables

**Table 1:** Participants’ BMI categories.

	PRE n (%)	POST n (%)
Underweight	2 (11.8)	-
Normal range	3 (17.6)	6 (35.3)
Overweight/pre-obesity	8 (47.0)	8 (47.0)
Obese class I	4 (23.5)	3 (17.6)

**Table 2:** Total and segmental body fat.

	PRE (mean ± sd)	POST (mean ± sd)	Paired Differences (mean ± sd)	95% CI	t	df	*p*-Value	r
Total Body Fat (%)	37.18 ± 7.08	35.46 ± 7.71	−1.72 ± 1.67	−2.57/ −0.86	4.24	16	0.001	0.73
Right Arm	37.22 ± 8.36	35.71 ± 8.53	−1.51 ± 1.47	−2.26/ −0.75	4.23	16	0.001	0.73
Left Arm	37.71 ± 7.93	36.38 ± 8.32	−1.34 ± 1.48	−2.10/ −0.58	3.73	16	0.002	0.68
Right leg	39.45 ± 5.49	38.24 ± 6.76	−1.21 ± 2.01	−2.24/ −0.18	2.49	16	0.000	0.53
Left leg	38.97 ± 5.93	37.59 ± 6.73	−1.38 ± 2.24	−2.53/ −0.23	2.54	16	0.024	0.54
Trunk	35.86 ± 8.15	33.78 ± 8.75	−2.09 ± 1.98	−3.10/ −1.07	4.35	16	0.022	0.74

**Table 3:** Participants’ fatigue measured by Modified Fatigue Impact Scale (MFIS).

	PRE (mean ± sd)	POST (mean ± sd)	Paired Differences (mean ± sd)	95% CI	t	df	*p*-Value	r
Overall fatigue	66.59 ± 9.27	52.59 ± 15.62	−14.00 ± 14.95	−21.69/ −6.31	3.86	16	0.001	0.69
Physical sub-scale	29.71 ± 4.91	21.06 ± 7.64	−8.65 ± 7.2	−12.35/ −4.94	4.94	16	0.000	0.78
Cognitive sub-scale	30.88 ± 4.57	27.24 ± 7.1	−3.65 ± 7.85	−7.68/ 0.39	1.91	16	0.073	-
Psychosocial sub-scale	6.00 ± 1.73	4.29 ± 2.26	−1.70 ± 1.76	−2.61/ −0.80	4.00	16	0.001	0.71

## Data Availability

The original contributions presented in this study are included in the article. Further inquiries can be directed to the corresponding author.
